# Infectious agents present in monk parakeet (*Myiopsitta monachus*) and rose-ringed parakeet (*Psittacula krameri*) invasive species in the parks of Madrid and Seville, Spain

**DOI:** 10.3389/fvets.2023.1162402

**Published:** 2023-08-07

**Authors:** Juan López, María Mogedas, Carlos Ballesteros, Bárbara Martín-Maldonado, Irene Sacristán, Raúl García, Juan Carlos Ortiz, Fernando Esperón

**Affiliations:** ^1^Veterinary Faculty, Universidad Alfonso X El Sabio Avenida de la Universidad 1, Villanueva de la Cañada, Madrid, Spain; ^2^Servicio de Consultoría para la Recuperación y Ordenación de la Fauna y su Ambiente S. L. (SCROFA), Madrid, Spain; ^3^Veterinary Department, School of Biomedical and Health Sciences, Universidad Europea de Madrid, Villaviciosa de Odón, Madrid, Spain; ^4^Centro de Investigación en Sanidad Animal (SIC-INIA), Valdeolmos, Spain; ^5^Departamento de Fauna y Biodiversidad, Área de Gobierno de Medio Ambiente, Ayuntamiento de Madrid, Madrid, Spain

**Keywords:** *Myiopsitta monachus*, one health, *Psittacula krameri*, real time PCR, urban parks, zoonotic pathogens, invasive species

## Abstract

**Introduction:**

The introduction of invasive species into an ecosystem could result in biodiversity loss and the spread of infectious agents that could cause re-emergent or emergent zoonotic diseases. Monk parakeets (*Myiopsitta monachus*) and rose-ringed parakeets (*Psittacula krameri*) are considered widespread invasive exotic species in urban habitats from the Iberian Peninsula. The aim of this study was to assess the presence of relevant infectious agents in wild parakeets captured in urban parks in Madrid and Seville (Spain).

**Methods:**

A total of 81 cloacal samples were collected and analysed using molecular techniques.

**Results:**

The prevalence of infectious agents varied between parakeet species: 9.5% of monk parakeets and 15% of rose-ringed parakeets were positive for enteropathogenic *E. coli* (EPEC), 13.3% of rose-ringed parakeets for avian influenza virus (AIV), 3.3% of rose-ringed parakeets for Newcastle disease virus (NDV), and a 23.8% of monk parakeets for *Chlamydia psittaci*.

**Discussion:**

All *C. psittaci*-identified isolates were classified as B, E, or E/B genotypes, indicating transmission from wild urban pigeons to parakeets. These results highlight the need for monitoring parakeet populations due to the implications for human and animal health.

## 1. Introduction

The introduction of invasive species into a new environment where they have never been before may have the same negative effects on the ecosystem as the introduction of exotic diseases, which can lead to biodiversity loss or even the extinction of local species (1). In this sense, numerous issues have been reported around the world, such as the loss of amphibian populations caused by *Batrachochytrium dendrobatidis*, a fungus carried by the African clawed frog (*Xenopus laevis*) (2), or the decline of the European red squirrel (*Sciurus vulgaris*) in the United Kingdom due to a poxvirus transmitted by the American grey squirrel (*Sciurus carolinensis*) (3). Moreover, invasive species can also lead to the emergence, or re-emergence, of zoonotic diseases (4). An example is the role of coypus (*Myocastor coypus*) in the spread of zoonotic *Leptospira* (5), or raccoons (*Procyon lotor*) as hosts for *Baylisascaris procyonis*, which causes neurological and ocular disease in humans (6).

Regarding the Aves Class, up to 971 introduced bird species have been reported in 230 countries (7). However, only a few studies confirmed the introduction of diseases by alien bird species, mostly restricted to sporadic cases (8). The most relevant was the historical exportation of feral pigeons (*Columba livia*) from the Mediterranean countries. This synanthropic species has been proven to carry more than 110 zoonotic pathogens (9); some of them in high proportions, such as *Campylobacter jejuni* and *Chlamydia psittaci* (10). Other alien species carrying zoonotic pathogens described in the scientific literature are the house sparrow (*Passer domesticus*), the common starling (*Sturnus vulgaris*), and the song thrush (*Turdus philomelos*) (8). Therefore, health status variables should be included when conducting foreign species risk assessments to evaluate the introduction of new agents and the changes in the epidemiology of existing ones (11).

In Spain, the estimated monk parakeet (*Myiopsitta monachus*) population was between 18,980 and 21,455 in 2016 (13), while the rose-ringed parakeet (*Psittacula krameri*) population ranged between 3,005 and 3,115 in 2015 (14). Since then, the populations of both species have largely increased. For example, in the capital, Madrid, the population of monk parakeets grew from 7,248–8,193 in 2015 to 11,154–12,975 in 2019 (15). Although both species are worldwide invasive gregarious Psittacidae, monk parakeets build communal nests that can be used by other species (13), which favours the diffusion, amplification, and spread of numerous pathogens, while rose-ringed parakeets do not. Instead, after the mating season, rose-ringed parakeets gather to roost reaching high-density flocks that pose the same hazard as communal nests. However, there is a lack of knowledge about the infectious agents that both invasive species can harbour.

In this context, the aim of this study was to assess the presence of zoonotic and loss biodiversity-related infectious agents in both species, namely monk parakeets and rose-ringed parakeets, from two densely populated Spanish cities.

## 2. Materials and methods

### 2.1. Samples and the areas of study

Due to the invasive ability of monk parakeets and rose-ringed parakeets, Spanish legislation includes an invasive species control program in which the removal of nests and euthanasia of trapped birds from these species are considered key to reducing populations (12).

First, the population size of each species in the different capture areas was established based on previous studies (16–19). Then, captures of monk parakeets in Madrid were performed between 2016 and 2017, while captures of rose-ringed parakeets in Sevilla took place between 2019 and 2020. Birds were trapped with both floor decoys with clap nets and nest traps. The sampling size (monk parakeets = 21 and rose-ringed parakeets = 60) was sufficient to detect a minimum expected prevalence of 13.2% and 4.8% in monk parakeets and rose-ringed parakeets, respectively for each analysed pathogen (Table 1) (www.winepi.net).

**Table 1:** Captured birds (*N*), number of analysed animals (*N* analysed), and percentage of animals analysed (%*N*) from the city council of Sevilla and Madrid, respectively.

**City**	**Species**	***N***	***N* analysed/% *N***	**Threshold prevalence**
Sevilla	*Psittacula krameri*	1,798	60/(3.3%)	4.8%
Madrid	*Myiopsitta monachus*	1,023–1,135	21/(1.9–2.1%)	13.2%

All the captured birds were checked by a veterinarian and sampled before euthanasia to obtain the cloacal content by enema, as described by Vázquez (10). In brief, 1 mL of sterile PBS was introduced into the cloaca using a sterile Pasteur pipette and immediately aspirated. The cloacal sample was transferred into a 2-ml microtube and diluted to a total volume of 2 ml to perform further analysis.

Handling procedures complied with European (Directive 2010/63/EU) and Spanish legislation (Royal Decree 53/2013). For sample collection, ethics approval was not necessary as samples were collected within the framework of a veterinary disease control intervention, and sampling was performed following standard procedural guidelines.

### 2.2. Pathogen detection

For each sample, RNA and DNA extraction were simultaneously performed from the cloacal enema using a pressure filtration method (QuickGene DNA Tissue Kit S, Fujifilm Life Science, Tokyo, Japan) and adding an RNA carrier (20).

Real-time PCRs (RT-PCRs) based on TaqMan^TM^ probes were performed for the partial amplification of the *inc*A gene of *Chlamydia psittaci* (21), the *map*A gene of *Campylobacter jejuni* (23), and the Prot6e gene of *Salmonella* spp. (26). Samples positive for *C. psittaci* were typed by RT-PCR based on Eva Green, with high-resolution melting (HRM) analysis (22), which amplifies a partial fragment (274 bp) of the *omp*A gene. Positive confirmation was sought by Sanger sequencing of the amplicons. In addition, real-time reverse transcriptase (RT-rtPCR) based on TaqMan^TM^ probes was performed to detect the avian influenza virus (AIV) matrix gene (24) and the Newcastle disease virus (NDV) matrix gene (25). Finally, zoonotic *E. coli* was detected following the protocol described previously for the detection of the intimin gene (*eae*A) (27, 28). Positive samples for the *eae*A gene were analysed to assess the presence of *stx-1* (29) and *stx-2* (28). If the sample was positive for at least one of them, it was considered enterotoxigenic (STEC) strain, but if the sample was negative for both genes, it was considered enteropathogenic (EPEC) (20, 27). Primers, probes, and methodology are summarised in Table 2.

**Table 2:** Methods used for determining infectious agents.

**Agent/gene**	**Method**	**Primers/probe**	**Annealing temperature (°C)**	**Reference**
AIV	RT-rtPCR (TaqMan probe)	5′-AGATGAGTCTTCTAACCGAGGTCG-3′ 5′-TGCAAAAACATCTTCAAGTCTCTG-3′ 5′-(6FAM)TCAGGCCCCCTCAAAGCCGA-BHQ1-3′	60	(24)
*C. jejuni*	rtPCR (TaqMan probe)	5′-CTGGTGGTTTTGAAGCAAAGATT-3′ 5′-CAATACCAGTGTCTAAAGTGCGTTTAT-3′ 5′-(6FAM)AATTCCAACATCGCTAATG-MGB-3′	60	(23)
*C. psittaci* (detection)	rtPCR (TaqMan probe)	5′-GCCATCATGCTTGTTTCGTTT-3′ 5′-CGGCGTGCCACTTGAGA-3′ 5′-(6FAM)TCATTGTCATTATGGTGATTCAGGA-MGB-3′	60	(21)
*C. psittaci* (genotyping)^a^	rtPCR (HRM-Eva Green)	5′-TGTGCAACTTTAGGAGCTGAGTTC-3′ 5′-GCTCTTGACCAGTTTACGCCAATA-3′	60	(22)
*E. coli* (*eae*A)	PCR	5′-TCAATGCAGTTCCGTTATCAGTT-3′ 5′-GTAAAGTCCGTTACCCCAACCTG-3′	55	(28)
*E. coli* (*stx*-1)^b^	rt PCR (Sybr Green)	5′-CATTACAGACTATTTCATCAGGAGGTA-3′ 5′-TCGTTCAACAATAAGCCGTAGATTA-3′	55	(29)
*E. coli* (*stx*-2)^b^	PCR	5′-CTTCGGTATCCTATTCCCGG-3′ 5′-CTGCTGTGACAGTGACAAAACGC-3′	55	(28)
NDV	RT-rtPCR (TaqMan probe)	5′-AGTGATGTGCTCGGACCTTC-3′ 5′-CCTGAGGAGAGGCATTTGCTA-3′ 5′-(6FAM)TTCTCTAGCAGTGGGACAGCCTGC-BHQ1-3′	60	(25)
*Salmonella* spp.	rtPCR (TaqMan probe)	5′-GTGAAATTATCGCCACGTTCGGGCAA-3′ 5′-TCATCGCACCGTCAAAGGAACC-3′ 5′-(6FAM)CTCTGGATGGTATGCCCGGTAAACA-BHQ1-3′	60	(26)

^a^Only applied in positive samples for *C. psittaci* detection.

^b^Only applied in positive samples for *eaeA* detection.

### 2.3. Statistical analysis

A non-parametric test (Mann–Whitney *U*-test) was applied to establish differences between monk parakeets and rose-ringed parakeets in the presence of each agent. Statistics were carried out using a commercially available software application (SPSS 29.0 software package; SPSS Inc., Chicago, IL, USA, 2002).

## 3. Results

In total, 81 birds were included in the study: 21 monk parakeets and 60 rose-ringed parakeets. Among monk parakeets, 23.8% were positive for *C. psittaci* (5/21; CI 95% 5.6–42.0%). The results of partial *omp*A gene sequencing revealed that positive samples could be classified as B, E, or E/B genotypes. No positive rose-ringed parakeets were detected for *C. psittaci* (*p* < 0.001). AIVs and NDVs were detected only in rose-ringed parakeets: 13.3% were positive for AIV (8/60: CI 95% 4.7–21.9%) and 3.3% for NDV (2/60; CI 95% 0.0–7.9%). Regarding *E. coli* presence, 9.5% of the monk parakeets were positive for the intimin (*eae*A) gene (2/21; CI 95% 0.0–22.1%), while the prevalence of this gene in rose-ringed parakeets was slightly higher (15%; 9/60; CI 95% 6.0–24%). All positive samples to *eae*A were analysed for *stx*-1 and *stx*-2 genes, all of which were negative and therefore enteropathogenic strains (EPEC). All samples were negative for *Campylobacter jejuni* and *Salmonella* spp. (Table 3).

**Table 3:** Results after analysis in search of the different infectious agents in both invasive species (*Myiopsitta monachus* and *Psittacula krameri*): number of positive birds, percentage from the analysed population for each agent, and interval confidence at 95% level.

**Infectious agents**	***Myiopsitta monachus***	***Psittacula krameri***
AIV	0	8 (13.3%; 95%CI: 4.7–22.0%)
*Chlamydophila psittaci*	5 (23.8%; 95%CI: 5.6–42.0%)	0
*Campylobacter jejuni*	0	0
EPEC	2 (9.5%; 95%CI: 0.0–22.1%)	9 (15.0%; 95%CI: 6.0–24.0%)
NDV	0	2 (3.3%; 95%CI: 0.0–7.9%)
*Salmonella* spp.	0	0

None of the birds showed clinical signs compatible with those pathogens, so positive birds were considered asymptomatic carriers.

## 4. Discussion

To the best of our knowledge, this is the first study of potentially zoonotic bacterial and viral agents present in invasive Psittacidae living in urban areas. The results demonstrate that invasive species, namely monk parakeets and rose-ringed parakeets, can host zoonotic pathogens such as *Chlamydia psittaci*, AIV, NDV, or EPEC.

### 4.1. Discussion about pathogens

#### 4.1.1. *Chlamydia psittaci*

The prevalence of *C. psittaci* detected in monk parakeets was in concordance with previous studies with captive individuals of the same species (30). However, although the results are similar, it is important to consider that the habitat conditions are not the same. In contrast to our results, *C. psittaci* was also described in rose-ringed parakeets in previous studies with different prevalence trends, but none of those studies employed specific PCR for *C. psittaci* detection (31, 32). Among the *Chlamydia* genotypes described for birds, all authors agree that genotype A is the most prevalent in Psittacidae (33, 34), while genotypes B, E, and E/B are more common in pigeons (35–37). To the best of our knowledge, this is the first *C. psittaci* genotyping in monk parakeets worldwide. The presence of genotypes B, E, or E/B in monk parakeets suggests that bacteria have been transmitted from pigeons to parakeets in the opposite direction than expected. The different prevalence trends between monk and rose-ringed parakeets could be due to the feeding behaviour of each species and, thus, their interactions with other species. While monk parakeets feed mostly on the ground (38), rose-ringed parakeets do so in trees more frequently (39). Feeding on the ground gives monk parakeets the chance to interact with feral pigeons and then share pathogens with them, mainly respiratory ones. *C. psittaci* is highly prevalent in pigeons from Madrid (10), magnifying the transmission between pigeons and monk parakeets. To confirm this hypothesis, several approaches could be attempted. One approach could be to demonstrate the presence of *C. psittaci* in pigeons from Seville. The other approach could be to genotype positive samples from pigeons. In this sense, previously unpublished results obtained by our research group confirmed the presence of genotypes B, E, and B/E in pigeons from Madrid, which are those found in the present study, supporting this hypothesis. However, a strong phylogenetic analysis such as sequencing the full *omp*A gene is required to demonstrate this potential transmission.

#### 4.1.2. Avian influenza virus

Information about avian influenza in monk and rose-ringed parakeets is scarce. To the best of our knowledge, the present study is the first one conducted on urban free-living birds. In monk parakeets, only one study assessed the presence of AIV by hemagglutination assay in a monk parakeet imported to Austria with a negative result (47). In rose-ringed parakeets, two positive captive birds for H9N2 strains were reported during routine virologic diagnosis of the birds imported to Japan (48). Unfortunately, in our study, assays for the identification of highly pathogenic AIV (i.e., H5 or H7 variants) could not be attempted due to the limited amount of sample.

#### 4.1.3. Newcastle disease virus

Similarly, the literature on NDV in monk and rose-ringed parakeets is old, and studies were performed using serology assays; therefore, positive samples only confirmed the contact of animals with the virus (49). Other studies focused on Psittacidae showed negative results for NDV detection (50, 51), with the exception of the study of NDV prevalence in India (52), where they found two positive samples out of four Psittaciformes analysed. Unfortunately, no data on which species were analysed were available. The present study confirms the presence of NDV in two rose-ringed parakeets. Unfortunately, the identification of velogenic strains could not be attempted due to the lack of a sample. Both AIV and NDV are notifiable in aviculture due to their serious economic and health repercussions, and wild birds, such as feral pigeons or hybrid ducks (*Anas* spp.), are considered reservoirs of both viruses (10).

#### 4.1.4. Escherichia coli

Diarrheagenic *E. coli* (DEC) is one of the main causes of human diarrhoea, and wild birds have been suggested as potential reservoirs for these pathogens (45). Only few articles about DEC detection in monk and rose-ringed parakeets have been published. In 1978, Graham and Graham could not find *E. coli* in the faeces of seven captive monk parakeets by bacteriological culture (46). Our results showed that 9.5% of monk parakeets and 15% of rose-ringed parakeets were positive for EPEC, but none of the strains was classified as STEC. Although there is a disparity, it is hardly comparable because of the publication date, the technique used, and the bird habitat. This represents the first description of EPEC in those Psittacine species.

#### 4.1.5. Campylobacter jejuni and Salmonella spp.

Finally, only few studies about *Campylobacter jejuni* and *Salmonella* spp. presence have been carried out in monk or rose-ringed parakeets (40–44). Our negative results agree with those published before for both species. However, a study performed on ring-rose parakeets confirmed 67% positivity for *Campylobacter* spp. with PCR detection (41).

### 4.2. General discussion

It is important to highlight that the gregarious behaviour of each species contributes to the spread of pathogens through their ecosystem. Moreover, co-infection has been observed in two rose-ringed parakeets, one of which is positive for AIV and EPEC and the other for AIV and NDV. In conclusion, the present study focuses on pathogens with potential zoonotic effects present in two invasive Psittacidae species and provides an approach to assess their health risk in the ecosystem. The increase of their populations in urban green zones could represent a hazard to both humans and biodiversity due to their role as reservoirs of zoonotic pathogens. In this context, our results highlight the need for surveillance and monitoring programs for these species.

## Data availability statement

The original contributions presented in the study are included in the article/supplementary material, further inquiries can be directed to the corresponding author.

## Ethics statement

The animal study was reviewed and approved by Universidad Alfonso X El Sabio. Institutional Ethics Committee approval Reference Number: 2023_3/181.

## Author contributions

JL: experimental design and text writing. MM: style and text revision. CB: sampling and text revision. BM-M and IS: sample lab analysis, results, and statistics. RG: sampling. JO: general revision. FE: experimental design, sample lab analysis, results, and statistics. All authors contributed to the article and approved the submitted version.

## References

[1] Bellard C Bernery C Leclerc C. Looming extinctions due to invasive species: Irreversible loss of ecological strategy and evolutionary history Running title: Functional and phylogenetic extinctions due to biological invasions. Glob Chang Biol. (2021) 27:4967–79. 10.1111/gcb.15771 34337834

[2] Stuart SN Chanson JS Cox NA Young BE Rodrigues ASL Fischman DL. Status and trends of amphibian declines and extinctions worldwide. Science. (2004) 306:1783–6. 10.1126/science.1103538 15486254

[3] Sainsbury AW Deaville R Lawson B Cooley WA Farelly SSJ Stack MJ. Poxviral disease in red squirrels *Sciurus vulgaris* in the UK: spatial and temporal trends of an emerging threat. Ecohealth. (2008) 5:305–16. 10.1007/s10393-008-0191-z 18923872

[4] Hulme PE. One Biosecurity: A unified concept to integrate human, animal, plant, and environmental health. Emerg Top Life Sci Portland Press Ltd. (2020) 4:539–49. 10.1042/ETLS20200067 33111945 PMC7803345

[5] Michel V Ruvoen-Clouet N Menard A Sonrier C Fillonneau C Rakotovao F. Role of the coypu (*Myocastor coypus*) in the epidemiology of leptospirosis in domestic animals and humans in source. Eur J Epidemiol. (2001) 17:111–21. 10.1023/A:1017931607318 11599683

[6] Popiołek M Szczęsna-Staśkiewicz J Bartoszewicz M Okarma H Smalec B Zalewski A. Helminth parasites of an introduced invasive carnivore species, the raccoon *(Procyon lotor L)*, from the warta mouth national park (Poland). J Parasitol. (2011) 97:357–60. 10.1645/GE-2525.1 21506875

[7] Dyer EE Redding DW Blackburn TM. The global avian invasions atlas, a database of alien bird distributions worldwide. Sci Data. (2017) 4:170041. 10.1038/sdata.2017.41 28350387 PMC5369319

[8] Roy HE Tricarico E Hassall R Johns CA Roy KA Scalera R. The role of invasive alien species in the emergence and spread of zoonoses. Biol Invasions. (2022) 25:1249–64. 10.1007/s10530-022-02978-1 36570096 PMC9763809

[9] Mia MM Hasan M Hasnath MR. Global prevalence of zoonotic pathogens from pigeon birds: a systematic review and meta-analysis. Heliyon. (2022) 8:e09732. 10.1016/j.heliyon.2022.e09732 35756122 PMC9218837

[10] Vázquez B Esperón F Neves E López J Ballesteros C Muñoz MJ. Screening for several potential pathogens in feral pigeons (*Columba livia*) in Madrid. Acta Vet Scand. (2010) 52:45. 10.1186/1751-0147-52-45 20569487 PMC2898782

[11] Andersen MC Adams H Hope B Powell M. Risk assessment for invasive species. Risk Anal. (2004) 24:787–93. 10.1111/j.0272-4332.2004.00478.x 15357799

[12] BOE-A-2013-8565. Disposición 8565 del BOE núm. 185 de 2013. Madrid: ED. Spanish State (2013).

[13] Molina B Postigo JL Muñoz AR del Moral JC. La Cotorra Argentina en España, población reproductora en 2015 y método de censo. Dissertation. Madrid: ED. SEO/BirdLife (2016).

[14] del Moral JC Somoza A Muñoz AR Molina B. La cotorra de Kramer en España, población reproductora en 2015 y método de censo. Dissertation. Madrid: ED. SEO/BirdLife (2017).

[15] Nebreda A Escudero E del Moral JC. Censo de Cotorra Argentina en el municipio de Madrid. Dissertation. Madrid: ED. SEO/BirdLife (2019).

[16] López R Ballesteros C López J. Memoria final sobre el inventariado y evaluación de nidos de Cotorra Argentina (Myopsitta monachus) en el Parque de las Cruces. Dissertation. Madrid: ED. SCROFA (2016).

[17] López R Ballesteros C López J. Memoria final sobre el inventariado y evaluación de nidos de Cotorra Gris Argentina (Myopsitta monachus) en el Parque del Oeste. Dissertation. Madrid: ED. SCROFA (2016).

[18] López R Ballesteros C López J. Memoria final sobre el inventariado y evaluación de nidos de Cotorra Argentina (Myopsitta monachus) en los jardines del Campo del Moro. Dissertation. Madrid: ED. SCROFA (2016).

[19] López R Ballesteros C López J. Informe sobre la estima poblacional de la Cotorra de Kramer (Psittacula krameri) en el Parque de María Luisa (Sevilla)-Dormidero Avenida de Borbolla. Dissertation. Sevilla: ED. SCROFA (2019).

[20] Sacristán C Esperón F Herrera-León S Iglesias I Neves E Nogal V. Virulence genes, antibiotic resistance and integrons in *Escherichia coli* strains isolated from synanthropic birds from Spain. Avian Pathol. (2014) 43:172–5. 10.1080/03079457.2014.897683 24689431

[21] Ménard A Clerc M Subtil A Mégraud F Bébéar C De Barbeyrac B. Development of a real-time PCR for the detection of *Chlamydia psittaci* [2]. J Med Microbiol. (2006) 55:471–3. 10.1099/jmm.0.46335-0 16533998

[22] Mitchell SL Wolff BJ Thacker WL Ciembor PG Gregory CR Everett KDE. Genotyping of *Chlamydophila psittaci* by real-time PCR and high-resolution melt analysis. J Clin Microbiol. (2009) 47:175–81. 10.1128/JCM.01851-08 19005152 PMC2620869

[23] Best EL Powell EJ Swift C Grant KA Frost JA. Applicability of a rapid duplex real-time PCR assay for speciation of *Campylobacter jejuni* and *Campylobacter coli* directly from culture plates. FEMS Microbiol Lett. (2003) 229:237–41. 10.1016/S0378-1097(03)00845-0 14680705

[24] Spackman E Senne DA Myers TJ Bulaga LL Garber LP Perdue ML. Development of a real-time reverse transcriptase PCR assay for type A influenza virus and the avian H5 and H7 hemagglutinin subtypes. J Clin Microbiol. (2002) 40:3256–60. 10.1128/JCM.40.9.3256-3260.2002 12202562 PMC130722

[25] Wise MG Suarez DL Seal BS Pedersen JC Senne DA King DJ. Development of a real-time reverse-transcription pcr for detection of newcastle disease virus RNA in clinical samples. J Clin Microbiol. (2004) 42:329–38. 10.1128/JCM.42.1.329-338.2004 14715773 PMC321685

[26] Malorny B Bunge C Helmuth R A. real-time PCR for the detection of *Salmonella Enteritidis* in poultry meat and consumption eggs. J Microbiol Methods. (2007) 70:245–51. 10.1016/j.mimet.2007.04.013 17512995

[27] Torres-Mejía AM Blanco-Peña K Rodríguez C Duarte F Jiménez-Soto M Esperón F. Zoonotic agents in Feral pigeons (Columba livia) from Costa Rica: possible improvements to dominish contagion risks. Vector Borne Zoonotic Dis. (2018) 18:49–54. 10.1089/vbz.2017.2131 29243991

[28] Vidal R Vidal M Lagos R Levine M Prado V. Multiplex PCR for diagnosis of enteric infections associated with diarrheagenic *Escherichia coli*. J Clin Microbiol. (2004) 42:1787–9. 10.1128/JCM.42.4.1787-1789.2004 15071051 PMC387562

[29] Chui L Couturier MR Chiu T Wang G Olson AB McDonald RR. Comparison of Shiga toxin-producing *Escherichia coli* detection methods using clinical stool samples. J Mol Diagn. (2010) 12:469–75. 10.2353/jmoldx.2010.090221 20466837 PMC2893631

[30] Origlia JA Cadario ME Frutos MC López NF Corva S. Detection and molecular characterization of Chlamydia psittaci and Chlamydia abortus in psittacine pet birds in Buenos aires province, Argentina. Rev Argent Microbiol. (2019) 51:130–5. 10.1016/j.ram.2018.04.003 30017323

[31] Pisanu B Laroucau K Aaziz R Vorimore F Gros A le Chapuis JL. Chlamydia avium detection from a ring-necked parakeet (Psittacula krameri) in France Chlamydia avium detection from a ring-necked parakeet (Psittacula krameri). J Exot Pet Med. (2018) 27:68–74. 10.1053/j.jepm.2018.02.035

[32] Chahota R Katoch RC Batta MK. Prevalence of *Chlamydia psittaci* among feral birds in himachal Pradesh, India. J Appl Anim Res. (1997) 12:89–94. 10.1080/09712119.1997.9706190

[33] Sutherland M Sarker S Vaz PK Legione AR Devlin JM Macwhirter PL. Disease surveillance in wild Victorian cacatuids reveals co-infection with multiple agents and detection of novel avian viruses. Vet Microbiol. (2019) 235:257–64. 10.1016/j.vetmic.2019.07.012 31383310

[34] Vanrompay D Butaye P Sayada C Ducatelle R Haesebrouck F. Characterization of avian *Chlamydia psittaci* strains using omp1 restriction mapping and serovar-specific monoclonal antibodies. Res Microbiol. (1997) 148:327–33. 10.1016/S0923-2508(97)81588-4 9765811

[35] Andersen AA. Serotyping of *Chlamydia psittaci* isolates using serovar-specific monoclonal antibodies with the microimmunofluorescence test. J Clin Microbiol. (1991) 29:707–11. 10.1128/jcm.29.4.707-711.1991 1890172 PMC269857

[36] Sachse K Laroucau K Vanrompay D. Avian chlamydiosis. Curr Clin Microbiol Rep. (2015) 2:10–21. 10.1007/s40588-014-0010-y

[37] Stokes HS Berg ML Bennett ATD A. Review of chlamydial infections in wild birds. Pathogens. (2021) 10:948. 10.3390/pathogens10080948 34451412 PMC8398480

[38] Postigo JL Carrillo-Ortiz J Domènech J Tomàs X Arroyo L Senar JC. Dietary plasticity in an invasive species and implications for management: the case of the monk parakeet in a Mediterranean city. Anim Biodivers Conserv. (2021) 44:185–94. 10.32800/abc.2021.44.0185

[39] Fraticelli F. The rose-ringed parakeet *Psittacula krameri* in a urban park: demographic trend, interspecific relationships and feeding preferences (Rome, central Italy). Avocetta. (2014) 38:23–8.

[40] De Luca C Niero G Cattarossi D Bedin M Piccirillo A. Pet and captive birds as potential reservoirs of zoonotic bacteria. J Exot Pet Med. (2018) 27:17–20. 10.1053/j.jepm.2017.10.01731383310

[41] Seifi S Khoshbakht R Azizpour A Seifi S. Occurrence of Campylobacter, Salmonella, and Arcobacter in pet birds of northern Iran. J Hellenic Vet Med Soc. (2019) 17:70. 10.12681/jhvms.22248

[42] Lopes ES Cardoso WM Albuquerque ÁH Teixeira RSC Salles RPR Bezerra WGA. Isolation of *Salmonella spp*. in captive Psittaciformes from zoos and a commercial establishment of Fortaleza, Brazil. Arq Bras Med Vet Zootec. (2014) 66:965–8. 10.1590/1678-41626643

[43] Gonzalez GA. Estudio serológico de Chlamidia psittaci y Salmonella sp., Virus Pox Aviar, Adenovirus y virus polioma en aves del orden psittaciforme en cautiverio en Dissertation. Chile: Universidad de Chile (2006).

[44] Allgayer MC Lima-Rosa CA Weimer TA Rodenbusch CR Pereira RA Streck AF. Molecular diagnosis of Salmonella species in captive psittacine birds. Vet Rec. (2008) 162:816–9. 10.1136/vr.162.25.816 18567929

[45] Lopes ES Maciel WC Medeiros PHQS Bona MD Bindá AH Lima SVG. Molecular diagnosis of diarrheagenic *Escherichia coli* isolated from *Psittaciformes* of illegal wildlife trade. Pesqui Vet Bras. (2018) 38:762–6. 10.1590/1678-5150-pvb-5083

[46] Graham CL Graham DL. Occurrence of *Escherichia coli* in feces of psittacine birds. Avian Dis. (1978) 22:717–20. 10.2307/1589649 373742

[47] Stunzner D Thiel W Potsch F Sixl W. Isolation of influenza viruses from exotic and central european birds. Zentralbl Bakteriol Mikrobiol Hyg. (1980) 247:8–17. 10.1016/S0172-5599(80)80015-0 7434995

[48] Mase M Imada T Sanada Y Etoh M Sanada N Tsukamoto K. Imported parakeets harbor H9N2 influenza A viruses that are genetically closely related to those transmitted to humans in Hong Kong. J Virol. (2001) 75:3490–4. 10.1128/JVI.75.7.3490-3494.2001 11238878 PMC114145

[49] Vijayan V. Role of Parrots in the Epizootiology of Newcastle Disease. Thesis. Mannuthy: College of Veterinary and Animal Sciences, Mannuthy (1981).

[50] Gilardi KVK Lowenstine LJ Gilardi JD Munn4 CA A. Survey for selected viral, Chlamydial, and parasitic diseases in wild dusky-headed parakeets (*Aratinga weddelii*) and tui parakeets (*Brotogeris Sanctithomae*) in Peru. J Wildl Dis. (1995) 31:523–8. 10.7589/0090-3558-31.4.523 8592384

[51] Johnson DC Couvillion CE Pearson JE. Failure to demonstrate viscerotropic velogenic newcastle disease in psittacine birds in the Republic of the Philippines. Avian Dis. (1986) 30:813–5. 10.2307/1590590 3814018

[52] Bansal N Singh R Chaudhary D Mahajan NK Joshi VG Maan S. Prevalence of newcastle disease virus in wild and migratory birds in Haryana, India. Avian Dis. (2022) 66:141–7. 10.1637/aviandiseases-D-21-00115 35510471

